# The Anterior GRadient (AGR) family proteins in epithelial ovarian cancer

**DOI:** 10.1186/s13046-021-02060-z

**Published:** 2021-08-27

**Authors:** Delphine Fessart, Jacques Robert, Cecile Hartog, Eric Chevet, Frederic Delom, Guillaume Babin

**Affiliations:** 1 INSERM U1242, “Chemistry, Oncogenesis Stress Signaling”, Université Rennes 1, Rennes, France; 2 Centre de Lutte Contre le Cancer Eugène Marquis, Rennes, France; 3 ARTiSt group, Univ. Bordeaux, INSERM, Institut Bergonié, ACTION, U1218, F-33000 Bordeaux, France

**Keywords:** Epithelial ovarian Cancer, Endoplasmic reticulum proteins, AGR2, AGR3

## Abstract

Epithelial ovarian cancer (EOC) is the most common gynecologic disorder. Even with the recent progresses made towards the use of new therapeutics, it still represents the most lethal gynecologic malignancy in women from developed countries.

The discovery of the anterior gradient proteins AGR2 and AGR3, which are highly related members belonging to the protein disulfide isomerase (PDI) family, attracted researchers’ attention due to their putative involvement in adenocarcinoma development. This review compiles the current knowledge on the role of the AGR family and the expression of its members in EOC and discusses the potential clinical relevance of AGR2 and AGR3 for EOC diagnosis, prognosis, and therapeutics.

A better understanding of the role of the AGR family may thus provide new handling avenues for EOC patients.

## Background

Despite many years of research, Epithelial Ovarian Cancer (EOC) remains the leading cause of death among gynaecological malignancies. This is mainly due to the absence of alarming symptoms, leading to late diagnosis, and to the lack of reliable screening methods allowing its early detection [1]. Nowadays, the standard treatment for ovarian cancer consists of an association of surgery, chemotherapy and targeted therapies [2]. In early-stages of ovarian cancer (limited to the ovaries or to the pelvic area), the first line of treatment consists of a surgery removing ovaries, with the fallopian tubes and the uterus. In advanced-stages of ovarian cancer, the surgery is completed with six cycles of chemotherapy regimen such as platinum-derived agent. Most of patients are diagnosed with advanced-stage of ovarian cancer, with many peritoneal implants, named peritoneal carcinomatosis. Thus, the treatment depends on the spread of peritoneal metastases and consists of primary cytoreductive surgery (also known as “debulking”) followed by platinum-based adjuvant chemotherapy. If non-resectable disease is found at preoperative imaging, the treatment is 3 to 4 cycles of neoadjuvant platinum-based chemotherapy followed by debulking surgery. After surgery, the treatment is completed with platinum-based adjuvant chemotherapy associated with targeted therapies. Carboplatin-paclitaxel chemotherapy combination is the standard first-line treatment of advanced ovarian cancer. For patients having high initial tumour load, Bevacizumab (a recombinant humanized monoclonal IgG1 antibody that targets vascular endothelial growth factor-A (VEGF-A)) is used. Recently, the implementation of PARP (Poly(ADP-Ribose) Polymerase) inhibitors in the therapeutic armamentarium has improved the prognosis of this malignancy. Although this success pledges for effective targeted therapies in EOC, this treatment is only effective in a small number of patients, essentially those bearing homologous recombination deficiencies (HRD) [3]. Therefore, the identification of proteins responsible for EOC development and progression is critical for both early detection and development of novel therapeutic approaches.

Since 2004, a new classification of EOCs has been developed [4, 5], dividing EOC into two categories, namely type 1 and type 2 (Table 1). Type 1 EOC includes different histological subtypes: endometrioid, clear cell, mucinous, sero-mucinous, and low grade serous. They usually present as large, unilateral, cystic neoplasms; except for clear cell carcinoma, these are low-grade, indolent malignancies with poor sensitivity to chemotherapy. They account for 10% of EOC deaths and exhibit a very good prognosis as long as they are confined to the ovary, a situation allowing complete cure using surgical treatment. Type 2 EOC are much more aggressive and account for 90% of EOC-related deaths. Type 2 ovarian tumour volume is generally lower than that observed in type 1 tumours, but the extraovarian disease is generally more important, with frequent spreading in the omentum and mesentery. The main histological subtype of type 2 EOS is high-grade serous carcinoma (HGSC), the other subtypes being undifferentiated carcinoma and carcinosarcoma. The poor outcome of type 2 EOC is due to the rapid evolution of the disease and the lack of early detection. Hence, they represent more than 75% of cases in advanced stage.

**Table 1:** Characteristics of the different types of EOC

	Type 1	Type 2
Anatomopathological subtype	Low grade serous Endometrioid Clear cells Mucinous tumour	High grade serous Carcinosarcoma Undifferentiated
Severity	10% of deaths	90% of deaths
Mutations	*ERRB2*, *KRAS*, *BRAF* pathway, MMR, *ARID1A*, *PTEN*, PI3K	P53, homologous recombination defects
Precursors	Borderline tumours, endometriosis	Serous Tubal In situ Carcinoma

On a molecular basis, the two types of EOC are very different (Table 1). Type 2 EOCs exhibit high genomic instability, p53 mutations and mutations in the homologous recombination repair (HRR) pathway, while type 1 EOCs display various mutations according to their histological subtype (*BRAF*, *KRAS*, *ARID1A*, etc.). Due to the biological and molecular differences among the histological EOC subtypes, it is now commonly preferred to consider each single subtype separately, discriminating between low and high grade serous tumours. Precursors and origin of EOC are still debated. While type 1 EOCs arise from precancerous lesions of the ovaries, HGSC is generally considered to arise from Fallopian tube, via a precursor called serous tubal in situ carcinoma (STIC). This is supported by growing evidence coming from the study of BRCA1/2 mutated patients undergoing prophylactic bilateral salpingo-oophorectomy [6]. Since BRCA1/2 mutated subjects present a high risk of developing EOC, prophylactic removal of ovaries and Fallopian tubes in these women decreases EOC risk. The analysis of post-operative samples showed no ovary lesions in these cancer-free patients, but dysplastic lesions arising in Fallopian tubes [4, 5].

Still, challenges in the management of ovarian cancer remains. So far, large scales trials lacked to find a proper screening tool allowing patients to be diagnosed at an early stage, with a good prognosis disease [7], and no preventive care is currently available, since only prophylactic bilateral salpingo-oophorectomy can be proposed to patients having high risk of developing the disease [1]. Many different inhibitors, such as tyrosine kinase inhibitors and monoclonal antibodies targeting cancer pathways, including angiogenesis, cell survival, cell growth, metastasis formation and DNA repair, are currently tested in clinical trials [8]. The most promising therapeutic agents include vascular endothelial growth factor (VEGF)-specific inhibitors and poly (ADP-ribose) polymerase inhibitors (PARPi). Although the success of PARPi pledges for effective targeted therapies in EOC, this treatment is only effective in a small number of patients, and the overall prognoses of advanced ovarian cancer is still poor in 2021. This is due in part by the heterogeneous nature of EOC and the lack of a common deregulated pathway in most patients. In addition, many publications have identified potential prognostic markers of EOC [9], however, most of these markers have an uncertain clinical value, their independent prognostic significance is unclear and none are used clinically [9]. Probably the main reason is a lack of reproducibility that can be explained by technical and biological factors. In addition, when biomarker data from cohorts with different proportions of subtypes are compared, the association of biomarkers with outcome or stage at presentation are rarely reproducible. No biomarker allowing an early diagnosis and no biomarker predictive of treatments’ effects is currently used in clinics. Therefore, the identification of proteins responsible for EOC development and progression is critical for both early detection and development of novel therapeutic approaches.

Gene expression signatures have recently shown that the expression of two highly related members of the protein disulphide isomerase family, the anterior gradient (AGR) proteins, AGR2 and AGR3, is deregulated in many tumours including EOC and that these proteins are involved in developmental processes and oncogenesis. In this article, we review the characteristics of the AGR protein family in EOC. We clarify the roles of AGR2 and AGR3 contributions to EOC and we discusses the potential use of these proteins as therapeutic targets and/or biomarkers in EOC.

## AGR family members

**Fig. 1:**
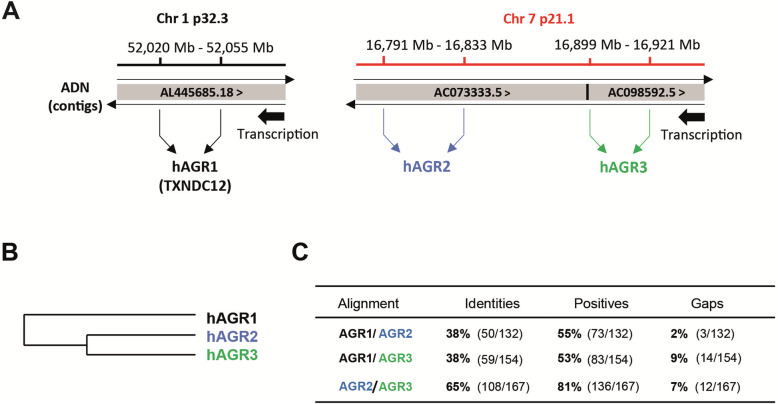
Comparisons of chromosomal gene positions and sequence homologies of AGR proteins. (**A**) Diagram representing the genomic position of the *TXNDC12* (AGR1), *AGR2* and *AGR3* genes respectively from left to right and their transcriptional orientation (Ensembl database, Human Genome GRCh38.13). (**B**) Phylogenetic tree (from Clustal Omega). (**C**) Protein sequence comparison of AGR proteins (Protein BLAST, https://blast.ncbi.nlm.nih.gov)

Anterior gradient genes were originally discovered in *X. laevis* and named AGR because of their expression patterns in the anterior region of the dorsal ectoderm during late gastrulation [10]. The human *AGR1* gene maps to chromosome band 1p32.3 whereas *AGR2* and *AGR3* genes map to chromosome band 7p21.3 (Fig. 1A). *AGR2* and *AGR3* are transcribed from the same DNA strand and are separated by only 60 kb of sequence. A phylogenetic analysis highlights the AGR distribution in two clusters: AGR1 group and AGR2/AGR3 group (Fig. 1B) showing that AGR2 and AGR3 are the most related. However, AGR proteins hardly differ in terms of size (172aa, 175aa and 166aa respectively for AGR1, AGR2 and AGR3) (Table 2) and molecular mass (19.206 kDa, 19.979 kDa and 19.171 kDa respectively for AGR1, AGR2 and AGR3) (Table 2). But, in terms of protein sequence homology, the alignment of AGR1, AGR2 and AGR3 sequences shows that AGR2 and AGR3 proteins are the most conserved (Fig. 1C). Indeed, the percentages of identical and similar amino acids are higher for AGR2/AGR3 proteins (65 and 81% respectively) than for AGR1/AGR2 (38 and 55%) or AGR1/AGR3 (38 and 53%) (Fig. 1C).

**Table 2:** Protein Comparison between the AGR family members

UniProt KB	Entry name	Protein name	Size (aa)	Mass (kDa)	Catalytic sequence	ER retention signal
O95881	TXD12_HUMAN	Thioredoxin domain-containing protein 12	172	19.206	CGHC	EDEL
O95994	AGR2_HUMAN	Anterior gradient protein 2 homolog	175	19.979	CPHS	KTEL
Q8TD06	AGR3_HUMAN	Anterior gradient protein 3	166	19.171	CQYS	QSEL

The human anterior gradient proteins, AGR1 (gene *TXNDC12*), AGR2 and AGR3, belong to the family of protein disulfide isomerases (PDI), whose function in the endoplasmic reticulum (ER) is to isomerise disulphide bonds in proteins acquiring their folded conformation [11]. In addition to their role in protein folding, several PDIs can act as molecular chaperones and participate to the ER-Associated Degradation pathway (ERAD). Outside of the ER, PDIs have been described as secreted proteins that function at the cell surface and the extracellular matrix [12–15] and have also been found in the cells’ cytosol [16]. The specific functions of the AGR family members as PDIs are poorly characterized. This could be explained by the fact that the consensus sequence for the thioredoxin catalytic motif is CXXC, and AGR2 and AGR3 have the particularity of lacking one cysteine in their active site motif (CXXS) (Table 2). This non-canonical catalytic motif (CXXS) in the thiol-reactive oxidative folding in vivo is unclear, although it is capable of forming the mixed S-S bonds with substrate proteins [17].

### AGR1

The first member of the AGR family, *AGR1* or *ERP18/19*, currently named *TXNDC12* (Thioredoxin Domain Containing 12), can be considered as the founding gene of the AGR family [18]. It presents high similarity with the classic thioredoxin fold containing CXXC motifs and is also found in invertebrates [19, 20]. The function of AGR1 involves its ability to form mixed disulphides with client proteins in the ER [21]. However, *AGR1* has never been identified as a cancer gene of interest in OMICS screens. So far only two articles have proposed AGR1 as a putative protein of interest in human cancer: one in gastric cancer, AGR1 would be involved in the promotion of cell growth, migration and invasion [22]; and the other in hepatocellular carcinomas, it would be involved in the promotion of Epithelial-Mesenchymal Transition (EMT) and metastasis [23]. Recently, Li N et al. [24] have identified a total of 102 hub molecules of proteins differentially expressed in EOC and one of them was AGR1; but its role remains unknown. Since AGR1 has been described, in different cancers, to be involved in the regulation of cell growth, migration, invasion, EMT and metastasis [22, 23]), it might play the same roles in EOC.

### AGR2

**Fig. 2:**
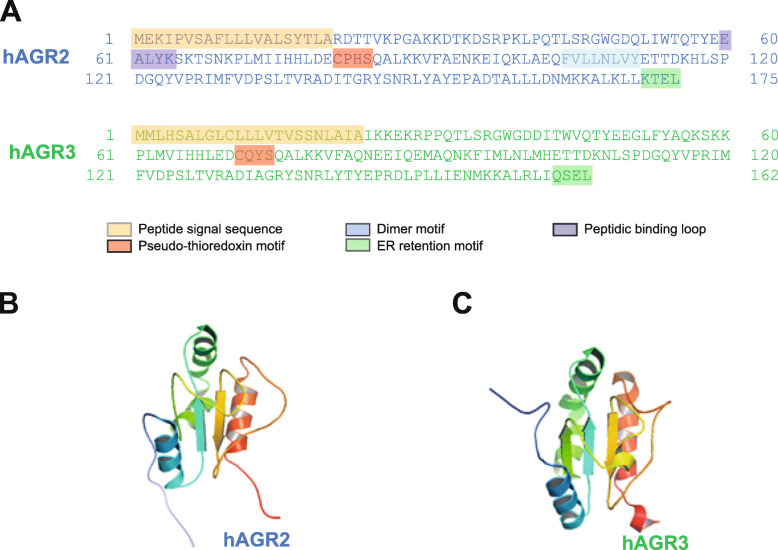
The functional domains of human AGR2 and AGR3 proteins and their 3D structure. (**A**) Primary sequences of human AGR2 and AGR3 and their different domains: Cleavable signal peptide, Pseudo-thioredoxin motif, Dimerization motif, Peptide binding loop, ER Retention signal. The sequences are extracted from the UniProtKB database (www.uniprot.org). The 3D structures of human AGR2 (**B**) and AGR3 (**C**) extracted from the RcsbPDB database (www.rcsb.org) and visualized using PyMOL software

Originally identified in *Xenopus,* anterior gradient-2 (XAG-2), a secreted cement gland-specific protein, has been described to play a role in the specification of the dorso-anterior ectoderm to cement gland and forebrain fates. In another amphibian model, the salamander, AGR2 was shown to allow limb regeneration from dedifferentiated and stem cells [25]. In human, AGR2 has been first identified in oestrogen-receptor-positive breast cancer cells [26]. Since this discovery, many studies reported the high expression of AGR2 in adenocarcinomas [27–29]. A meta-analysis established that AGR2 might be a potential biomarker of prognosis in solid tumour patients [30]. Although AGR2 is an ER-resident protein (endoplasmic reticulum AGR2 (erAGR2)) and harbours an ER retention signal sequence (KTEL) (Fig. 2A) (Table 2), AGR2 is secreted in the extracellular medium, blood or urine [28, 29, 31–34] and is also found in the cell cytosol [35]. Moreover, it has been shown that AGR2 exists not only as a monomer, but it can also form homodimers [36]. Indeed, AGR2 forms dimers through residues E60 and C81 [36]. Recently, we have shown that the AGR2 homodimer is the ER-resident form and the AGR2 monomer is the secreted extracellular form (extracellular AGR2 (eAGR2)) [37]. The 3D structures of the AGR2 and AGR3 proteins are close, except that the AGR2 protein has one β-sheet less (Fig. 2B-C). As yet, the functions by which AGR2 promotes carcinogenesis growth are poorly understood in EOC, but it has been demonstrated, in diverse adenocarcinomas, roles of AGR2 in different tumour-associated processes such as proliferation, migration, invasion and metastasis [27–29, 36–39]. Indeed, we and others have recently demonstrated an emerging role for eAGR2 in the tumour development, which clearly attest that eAGR2 protein acts as an extracellular regulator, through gain-of-extracellular functions, on phenotypes associated with tumour morphogenesis, tumorigenicity, and inflammation [27–29, 36, 38, 40]. AGR2 has been shown to participate in various cancer signalling pathways including Hippo, EGFR, EsR, cyclin D1, Src, c-Myc, survivin, aryl hydrocarbon receptor (AhR) and transforming growth factor-beta (TGF-β) [41]. Recently, we also showed that AGR2 is refluxed from the ER to the cytosol (cytosolic AGR2 (cAGR2)) and, in the latter compartment, acts, through a gain-of-cytosolic function, as an inhibitor of p53 tumour suppressor activity [27, 35]. Hence, AGR2 can stimulate tumour growth, invasion, resistance to chemotherapy and metastasis, in a wide range of human adenocarcinoma types. In an attempt to better explain the function of AGR2 in EOC, we raise the hypothesis that the deregulation of AGR2 localizations, cytosolic (cAGR2) and extracellular (eAGR2), could exert different pro-oncogenic gain-of-functions to confer to EOC specific and evolutive features. As a consequence, the regulation of AGR2 activities and localizations (erAGR2, cAGR2 and eAGR2) in EOC might determine tumour evolution in a way that would promote its growth and aggressiveness. Even if not yet investigated in EOC, gain-of-pro-oncogenic functions associated with shifts in these localizations, could contribute to the development and progression of EOC and could be targeted as a therapeutic strategy against EOC.

### AGR3

AGR3 was first identified in a proteomic analysis of purified membrane preparations from human breast adenocarcinoma cell lines [42]. Unlike AGR2, the functional roles of AGR3 in carcinogenesis remain unclear. AGR3 was reported to be overexpressed in breast [42], prostate [43], liver [44] and epithelial ovary cancers [45]. Although AGR3 also harbours an ER retention signal sequence (QSEL) (Fig. 2A) (Table 2), AGR3 is secreted in the extracellular medium or blood [34, 46]. Recently, we have demonstrated that extracellular AGR3 (eAGR3) has gain-of-extracellular pro-oncogenic functions within the extracellular space and could promote cancer aggressiveness. Indeed, eAGR3 regulates breast cancer cell migration via SRC signalling, and thus promotes the proliferative and invasive abilities of breast cancer cells [46]. AGR3’s role as a component of tumour signalling pathway remains poorly understood.

## AGR2 and AGR3 in EOC

### Expression and biological functions of AGR2 and AGR3

AGR2 expression level varies among various EOC cell lines. Indeed, undetectable expression was observed in cultures of human clear cell ES-2 carcinoma or in ES-2 xenograft tumours, whereas both cultured human ovarian carcinoma A2780 cells and A2780 xenograft tumours express significant amounts of AGR2. SK-OV-3 human ovarian carcinoma cells (derived from the ascitic fluid) display very low AGR2 expression level when cultured in vitro but produce large amounts of AGR2 during the generation of xenograft tumours in vivo. However, AGR2 expression is lost when tumour cells were re-isolated from the xenografted tumours and re-cultured in vitro [47]. It has also been shown that AGR2 is secreted from tumour ovarian cells (SK-OV-3 ovarian cancer cell line derived from ascites fluid) and accumulates in the tumour interstitial fluid in EOC xenograft tumours [47]. To generate a mouse model that would mimic human ovarian cancer metastasis, SK-OV-3 human ovarian cancer cells were injected into the perineum of nude mice [48]. In metastasised tumour tissues (peritoneum) of these mice, aberrant DNA methylation at CpG sites in the *AGR2* promoter region [48] was reported, and associated to an aggressive cell phenotype (migration and invasion). Moreover, the stable overexpression of AGR2 in human ovarian carcinoma cells (MDAH 2774, derived from ascitic fluid) enhanced cell growth and migration of MDAH 2774 cells [49]. Consistent with the involvement of AGR2 in cell growth and migration, a cDNA microarray analysis of these AGR2 stably overexpressing human MDAH 2774 ovarian cancer cells, revealed that AGR2 overexpression up-regulates the expression of genes involved in cell proliferation, invasion, and angiogenesis, which play a role in tumour progression and metastasis in EOC [49]. Since AGR2 has been reported to be associated with tumour progression and metastasis in several carcinomas [27, 28, 36], it would be interesting to decipher the role of AGR2 in ovarian tumour progression and metastasis. An in-depth analysis of the mechanisms regulating AGR2 expression in EOC and the functional consequences of the observed deregulation would provide information on its functional significance and its role in diagnosis.

AGR3 has been found to be overexpressed in four different types of primary human EOC: serous, endometrioid, clear cell (non-mucinous types) and mucinous carcinomas [50]. In a cohort of 415 ovarian tumour tissue samples among which 238 serous tumours, 38 endometrioid tumours, 51 clear cell tumours, 32 mucinous tumours and 56 other tumour subtypes, 204 samples stained positive for AGR3, with 90% strong expression, 10% moderate expression, and no or weak expression [51]. AGR3 expression patterns in serous and clear cell EOC tissues showed that AGR3 was highly expressed in serous EOC and in clear cell EOC in tumour versus adjacent tissues [51]. Gene expression of AGR3 in low grade ovarian serous carcinomas (LGSC), HGSC, and serous borderline tumours, showed that AGR3 is upregulated in LGSC and in serous borderline tumours as compared to regular ovarian surface, but not in HGSC [45]. These results were also confirmed by mRNA expression data from 5 HGSC and 6 LGSC, showing that AGR3 was upregulated in LGSC compared to HGSC [52]. These expression profiles were also observed by RT-PCR on 36 HGSC and 16 LGSC. Moreover, in the majority of LGSC cases, < 10% of the tumour cells exhibited a positive staining. On the other hand, HGSC specimens also exhibited rare AGR3 staining (20 positive samples out of 103). In conclusion, AGR3 is overexpressed in some EOCs. A question remains as to its specificity concerning one particular EOC subtype. Indeed, depending on the study, serous tumours were mixed with HGSC and LGSC and did not discriminate between these two subtypes. The biological roles and functions of AGR3 protein have not been extensively studied. It has only been reported that AGR3 mediates cisplatin resistance in ovarian tumour xenograft, suggesting that AGR3 is pro-oncogenic and exerts functions independently of AGR2 [50]. In conclusion, the role of AGR3 as a tumour signalling molecule in EOC is poorly understood and requires further investigation.

### The cooperative or antagonistic role of AGR2 and AGR3 in EOC

**Fig. 3:**
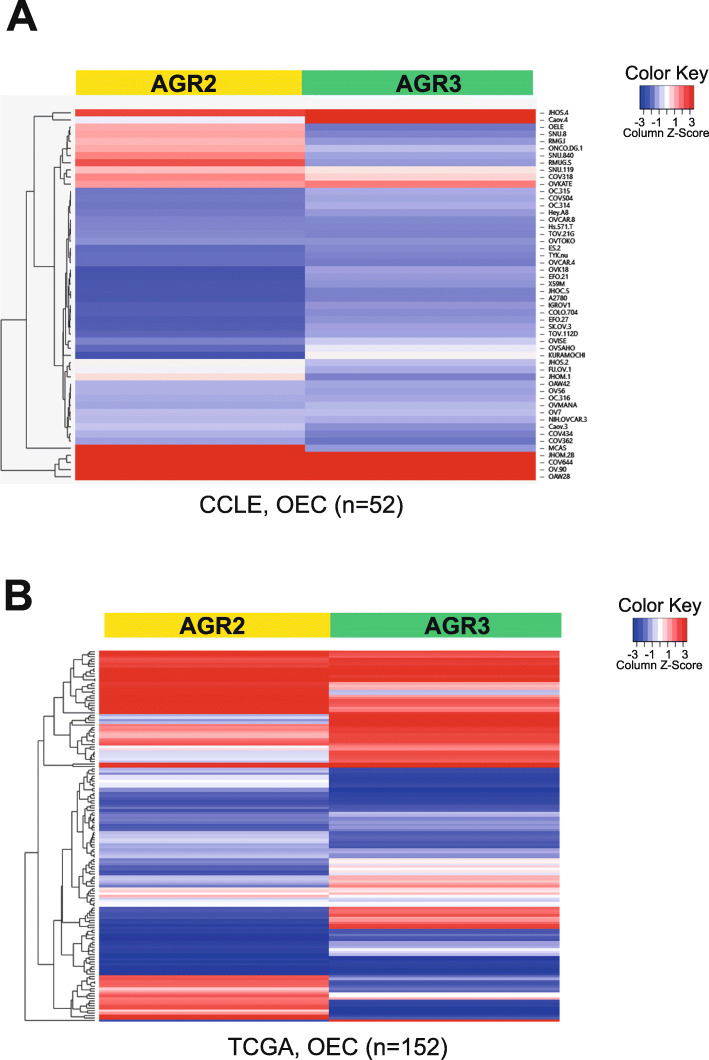
Uncoupling and coupling of AGR2 and AGR3 expression in human EOC databases Heatmaps of differential expression of AGR2 and AGR3 genes in EOC. Data shown are the relative expression level in ovarian cancer cell lines from CCLE database (*n* = 52) (**A**) and the relative expression level in ovarian cancer tissues from TCGA database (*n* = 152) (**B**)

AGR2 and AGR3 are co-expressed in human breast cancer [53], while the expression of AGR2 is exclusive to prostate cancer [54]. In a model of ovarian cancers, Gray et al. have described an uncoupled expression of both AGR proteins [50], it appears that AGR3 expression is associated to tumour type: in non-mucinous tumours, AGR3 expression is heterogeneous, oestrogen receptor-independent and not related to AGR2 expression, whereas in mucinous tumours, AGR2 and AGR3 stained both positive. Thus, both coupled and uncoupled expression of AGR2 and AGR3 proteins are described in EOC. However, it is the only report on AGR2/AGR3 co-expression in EOC. Therefore, using in silico analyses, we evaluated the expression of AGR2 and AGR3 mRNA in both the EOC cancer cell line encyclopedia (CCLE) (Fig. 3A) and the EOC cancer genome atlas (TCGA) (Fig. 3B) databases. These analyses showed that the expression of both AGR2 and AGR3 mRNA can be correlated or not in OEC. We propose that AGR2 and AGR3 could exert different pro-oncogenic functions to confer specific and evolutive features to these ovarian tumours. For instance it would be interesting to investigate whether the different expression patterns of AGR3 and AGR2 in mucinous and non-mucinous (serous, endometrioid and clear cell) ovarian carcinomas could be correlated with patient prognosis. Indeed, distinct pathogenetic pathways are believed to be involved in the differentiation of the subsets of ovarian carcinoma from ovarian epithelial cells. As a consequence, expression regulation of both AGR2 and AGR3 in EOC could determine tumour evolution in a way that would promote its growth and aggressiveness. Hence, cooperative or antagonistic biological effects could be expected, thus associating the differential expression of AGR2 and AGR3 to ovarian tumour outcomes. In this regard, pro-oncogenic functions associated with AGR2 and AGR3 expressions could be targeted as a therapeutic strategy in EOC. In conclusion, the co-expression of AGR2 and AGR3 in EOC is poorly understood, and thus warrants further investigation.

### AGR2 and AGR3 as diagnostic biomarkers

Biomarkers can serve multiple purposes in diagnosis thus we will review the different possible purpose of the AGRs in the diagnosis. Among EOCs, mucinous and endometrioid tumours express high levels of AGR2 (Table 3). Therefore, AGR2 expression is often associated with mucinous tumours and their precursors, namely mucinous cystadenoma and mucinous borderline tumours, reaching 62 to 100% expression levels for mucinous tumours and 35–89% for endometrioid tumours (Table 3). For comparison, AGR2 was found to be expressed in various proportions in HGSC (12 to 70%, depending on the cohort) (Table 3). Thus, AGR2 expression is variable, depending on tissue and on tumour type. We can speculate that AGR2 could be a useful tool to differentiate mucinous cancers from other EOC, and this would be consistent with the fact that a major characteristic of AGR2 expression is to be also representative: i) of other mucinous adenocarcinomas subtypes from different origins (breast, colon, gastric, lung, pancreas, stomach, ...) [30]; ii) of mucin-secreting cells [36]. However, this has to be experimentally demonstrated in EOC.

**Table 3:** AGR2 expression using IHC in different cohorts from literature

	Subtype	Armes et al. [43]	Edgel et al. [25]	Darb et al. [42]	Park et al. [37]	Alves et al. [44]
		Nb of positives samples/ Total Nb of samples				
**Surface epithelium**		11/36	0/5	/	/	/
**High grade serous**	Type 2	3/19	7/10	40/124	6/12	14/109
**Endometrioid**	Type 1	17/19	3/3	5/15	/	/
**Mucinous**	Type 1	15/15	/	5/8	4/4	/
**Low grade serous**	Type 1	1/2	/	4/16	/	/
**Clear cell**	Type 1	9/20	1/1	1/9	/	/

For AGR3, its expression has been reported to be increased in EOC tissue compared to adjacent tissue, suggesting that AGR3 protein might serve also as a diagnostic marker for EOC [47]. An mRNA expression profile from 5 HGSC and 6 LGSC has shown that *AGR3* mRNA was highly upregulated in LGSC compared to HGSC [48]. This difference in expression was confirmed by RT-PCR on 36 HGSC and 16 LGSC. In addition, comparing *TP53* and *AGR3* expressions in 8 HGSC and 7 LGSC revealed that *TP53* was high and AGR3 low in HGSC, whereas *TP53* was low and *AGR3* high in LGSC [48]. These results suggest that *TP53* and *AGR3* differential expression could be used for differential diagnosis of HGSC (*TP53* high / *AGR3* low) and LGSC (*TP53* low/*AGR3* high). Furthermore, an immunohistochemical analysis on 145 HGSC and 30 LGSC showed that low p53 protein expression with high AGR3 protein expression was significantly associated with LGSC with a sensitivity of 70% and a specificity of 97.9% [48]. Hence, combination of AGR3 and p53 protein expression might be useful to differentiate the different ovarian carcinomas subtypes. In conclusion, future prospective clinical studies conducted on a larger cohort of various ovarian carcinoma subtypes would validate the clinicopathological utility of AGR3, in combination with p53.

Some studies have also suggested that plasmatic AGR2 could serve as a follow-up biomarker in EOC. Indeed, eAGR2 protein has been detected in the serum of EOC patients which was reported in several studies [31, 49]. Park et al. [49] detected an increase in the mean eAGR2 levels in the serum of mucinous ovarian tumour patients and not in the serum of serous ovarian tumour patients (mean 13 ng/mL in mucinous tumours vs. mean 2 ng/mL in serous tumours and mean 4 ng/mL in normal subjects). In the same cohort study, they correlated the level of eAGR2 to mRNA AGR2 expression and found a 5-fold up-regulation in mucinous ovarian tumour patients. In another study, Edgel et al. [32] measured the plasma concentration of eAGR2 and CA125 in a cohort of 46 patients suffering from EOC, mainly composed of serous carcinoma patients (*n* = 36), as compared to 61 control cases. CA125 is a glycoprotein found in serum and is a tumour marker recommended for clinical use in the management of EOC. CA125 is especially useful in EOC to monitor treatment responses. Nonetheless, CA125 can be elevated in endometriosis or during menstruation as well; thus, its ability to detect an early EOC is poor [55]. The plasma concentration of AGR2 was found significantly higher in patients with EOC than in patients free from cancer. All ovarian tumour types analysed exhibited elevated the median circulating levels of AGR2 (750 pg/mL vs. 200 pg/mL), and both serous and non-serous cases of all stages presented significantly increased the median circulating levels of AGR2 (875 pg/mL in serous tumours, vs. 680 pg/mL in non-serous). When compared to CA125, this increase was present in non-serous tumours as well, whereas CA125 was elevated mainly in serous tumours. The authors proposed that a combination of CA125 and AGR2 would better discriminate EOCs from controls [32]. These findings were further confirmed using the same cohort by Rice et al. [56]. In addition to CA125 and AGR2, the authors compared the levels of the protein midkine (MDK). MDK is a pleiotropic growth factor prominently expressed during embryogenesis but down-regulated to negligible levels in healthy adults. Many studies have demonstrated striking MDK overexpression in various pathologies, including many cancers, as compared with healthy controls. Median plasma concentrations of immuno-reactive MDK, AGR2 and CA125 were significantly higher in the patient cohort (909 pg/ml, 765 pg/ml and 502 U/ml, respectively *n* = 46) than in the control cohort (383 pg/ml, 188 pg/ml and 13 U/ml, respectively *n* = 61) (*p* < 0.001). However, within control or patients cohorts, plasma concentrations of AGR2 displayed no significant correlations with either CA125 or MDK concentrations. No statistically significant effects of either tumour type or stage on biomarker plasma concentrations were identified [57]. Hence, a combined multi-analysis model could better discriminate patients and controls.

A third study within the UK Collaborative Trial of EOC Screening (UKCTOCS), encompasses 490 serial serum samples from 49 women later diagnosed with EOC and 31 control cancer-free women. UKCTOCS is a prospective trial in which different screening strategies were tested. Primary endpoint was the reduction in specific mortality by EOC according to the screening strategy. Patients had regular blood tests during the whole study, between 2001 and 2005, providing a longitudinal collection of serum samples. The authors selected different proteins to assess plasma concentrations before EOC diagnosis. They found that incorporating CA125, HE4, CHI3L1, PEBP4, and/or AGR2 provided 85.7% sensitivity at 95.4% specificity up to 1 year before diagnosis [58]. Overall, these studies led us to speculate that plasmatic AGR2 (eAGR2) might be a useful biomarker, to be used in combination, for EOC screening.

### AGR2 and AGR3 as prognostic biomarkers

In a series of 124 HGSCs, high cellular expression of AGR2 has been reported to be negatively correlated with patients’ survival [59]. The mean survival until progression was reduced from 38.71 months in patients with AGR2 low-expressing tumours to 12.38 months in patients AGR2 high-expressing tumours. The multivariate Cox regression analysis including patient age, FIGO stage, and residual tumour after surgery showed that high AGR2 expression was an independent prognostic marker, both for OS (hazard ratio (HR) = 2.60, *p* = 0.023) and PFS (HR = 3.89, *p* = 0.001) [59]. In another study from Armes et al. [60], with a mixed-cohort composed of surface epithelial EOC [36], HGSC [19], endometroid tumours [19], mucinous tumours [15], LG tumours [2], and clear cell carcinomas [20], it has been reported that among the 23 AGR2 negative patients, 17 (74%) had relapsed, with only six patients remaining disease-free at the last follow-up. In contrast, only 8 of 36 patients with AGR2 expression had relapsed (22%), with 28 disease-free patients at the last follow-up. In the study of Alves et al. [61] using a cohort composed of 167 patients with 109 HGSC, the median of disease-free survival of patients whose tumours presented AGR2 positivity was 44 months, vs. 22 months in case of negativity. The Cox proportional hazard regression model showed that the absence of AGR2 protein expression in the tumour was a strong predictor of poor disease-free survival (HR: 0.631; 95% confidence interval: 0.412–0.966; *P* = 0.034). In terms of prognosis, the fact that all studies had a low number of patients and mixed different types of EOC, which are known to have very different behaviour and prognosis, led us to the impossibility to conclude on the prognostic value of AGR2.

For AGR3, Samanta et al. [51] have observed that high AGR3 expression adversely affects overall survival in a mixed cohort of EOC patients. This is in opposition with the results of King et al. [45] on a cohort of 103 HGSC and 56 LGSC, who observed that higher AGR3 expression is associated with longer median survival in ovarian carcinoma. Again, these results may be explained by the heterogeneity of the patient populations studied. To understand the clinical outcome in relation to AGR3 and/or AGR2 expression, further studies are required to draw firm conclusions.

### AGR2 and AGR3, potential candidates for therapy targeting

A humanised monoclonal antibody has been developed to target extracellular AGR2 (eAGR2) and tested in mouse xenografts of the SK-OV-3 human ovarian carcinoma cell line (derived from ascitic fluid). The blocking antibody reduced xenograft growth by 50% compared to the control group [62]. The effect of eAGR2 blocking antibody on xenograft ovarian tumour growth from two other different ovarian tumour cell lines has also been investigated and showed that treatment reduced significantly the size of A2780 and SK-OV-3 xenograft ovarian tumours, which constitutively express AGR2 [47] whereas treatment had no effect on ES-2 xenograft ovarian tumours, which does not express AGR2. Moreover, eAGR2 blocking reduced the infiltration of vascular endothelial cells and fibroblasts in AGR2-positive ovarian tumours [47]. These results indicate that eAGR2 is a putative tumour target for the treatment of AGR2-positive EOC. In conclusion, eAGR2 possess several characteristics suitable for therapeutic antibody targeting in EOC, including **i**) extracellular localisation for antibody access, and **ii**) induced expression during tumour formation. Therefore, blocking monoclonal antibody against eAGR2 could be an effective anti-tumour monoclonal antibody and may have therapeutic potential against EOC.

For AGR3, Gray et al. [50] have investigated the expression of AGR3 in human ovarian cancer and identified a role for AGR3 in the resistance of the DNA-damaging agent cisplatin in a mouse xenograft model. Therefore, AGR3 could also represent a possible novel target to circumvent drug resistance in EOC. Nevertheless, this effect still needs to be confirmed in EOC.

## Future perspectives

The human AGR2 and AGR3 genes map to chromosome band 7p21.3, proteins are clustered together by phylogenetic analysis and share 65% sequence identity and 81% of sequence positive (Fig. 1A-C). Both are transcribed from the same DNA strand and are separated by a sequence of only 60 kb. Although the contiguity of AGR2 and AGR3 would suggest that they are co-regulated, the contrary can be observed in some EOC samples (Fig. 3A-B). Hence, the non-homology of key domains, of both AGR2 and AGR3 (Fig. 2A), offers a rare opportunity to evaluate the significance of minor structural changes on events central to ovarian tumour progression. Thus, we raise the hypothesis that the difference in the deregulation of AGR2 and AGR3 expression in EOC could yield specific cancer phenotypes, and could thus pave the way to an actionable therapeutic strategy.

Despite their heterogeneity, clinical management of all ovarian carcinoma subtypes is standardised and consists of a combination of radical surgery and chemotherapy with the cisplatin analogue carboplatin, either alone or in combination with paclitaxel, as well as PARP-Inhibitor treatment in BRCA mutant patients. Further knowledge of the sequence of molecular events involved in the differentiation of EOC subtypes should ultimately enable the development of targeted therapies. Patients with advanced mucinous ovarian cancer have a lower response to first-line chemotherapy as compared to patients with other histological subtypes, and drug resistance is therefore thought to be one of the main causes for the poorer prognosis of patients with mucinous ovarian cancer. As such, it has been suggested that AGR3 might contribute to cisplatin resistance. This indicates that AGR3 might represent a novel candidate to be characterized in order to reverse drug resistance in EOC. In other cancers, an interaction between AGR2 and the development of chemoresistance has been shown, suggesting that inhibition of AGR2 could be a potential strategy to overcome chemoresistance. Development of novel strategies to overcome chemoresistance is a central goal in EOC research. Therefore, AGR2 and AGR3 might represent attractive targets in chemoresistance whose function alteration could support or restore chemosensitivity.

The detection of eAGR2 and eAGR3 in patient’s serum might also represent a good strategy for ovarian tumour follow-up. However, validation of eAGR2 and eAGR3 as potential serum markers is essential and should be performed with a large-scale analysis of EOC blood samples and compared with other validated serum markers such as CA125 as potential biomarkers for patient follow-up or early screening of the disease.

## Conclusions

EOC represents a clinical model for evaluating the cooperative or antagonistic role of AGR2 and AGR3 in cancer and in drug resistance. Indeed, concomitant or opposed of AGR2 and AGR3 expression in EOC could determine tumour evolution in a way that would promote its growth and aggressiveness, thus associating the differential expression of AGR2 and AGR3 to tumour outcomes. The poor survival observed in EOC since nearly 20 years highlights the urgent need to discover new actors involved in the development and progression of this cancer. This is critical for both early detection and development of novel therapeutic approaches. In this regard, pro-oncogenic functions associated with AGR2 and AGR3 could be important to explain the mechanism of chemoresistance, to identify therapeutic targets and to develop new treatments. Moreover, the fact that most HGSC are diagnosed at an advanced stage with no reliable screening to date, AGR proteins as early biomarkers could bring innovating tools to combat EOC. In conclusion, our questions and hypotheses on AGRs must be further investigated at the clinical and basic science levels to have a real impact on the diagnosis, prognostic and therapeutic for ovarian cancer.

## Data Availability

Not applicable.

## Abbreviations

AGR: anterior gradient
EOC: epithelial ovarian cancer
PDI: protein disulfide isomerase
HRD: homologous recombination deficiencies
HGSC: high-grade serous carcinoma
HRR: homologous recombination repair
STIC: serous tubal in situ carcinoma
ER: endoplasmic reticulum
ERAD: ER-Associated Degradation pathway
EMT: Epithelial-Mesenchymal Transition
eAGR2: extracellular AGR2
eAGR3: extracellular AGR3
MDK: midkine
UKCTOCS: UK Collaborative Trial of EOC Screening
LGSC: low grade ovarian serous carcinomas
CCLE: cancer cell line encyclopedia
TCGA: the cancer genome atlas
